# Scale-up of nature’s tissue weaving algorithms to engineer advanced functional materials

**DOI:** 10.1038/srep40396

**Published:** 2017-01-11

**Authors:** Joanna L. Ng, Lillian E. Knothe, Renee M. Whan, Ulf Knothe, Melissa L. Knothe Tate

**Affiliations:** 1 Graduate School of Biomedical Engineering, University of New South Wales (UNSW) Australia, Sydney, Australia; 2 School of Art & Design, University of New South Wales (UNSW) Australia, Sydney, Australia; 3 Biomedical Imaging Facility, Mark Wainwright Analytical Centre, UNSW Australia, Sydney, Australia; 4 Cleveland Clinic, Cleveland, USA; 5 TissuTex Pty Ltd, Wentworth Falls, Australia

## Abstract

We are literally the stuff from which our tissue fabrics and their fibers are woven and spun. The arrangement of collagen, elastin and other structural proteins in space and time embodies our tissues and organs with amazing resilience and multifunctional smart properties. For example, the periosteum, a soft tissue sleeve that envelops all nonarticular bony surfaces of the body, comprises an inherently “smart” material that gives hard bones added strength under high impact loads. Yet a paucity of scalable bottom-up approaches stymies the harnessing of smart tissues’ biological, mechanical and organizational detail to create advanced functional materials. Here, a novel approach is established to scale up the multidimensional fiber patterns of natural soft tissue weaves for rapid prototyping of advanced functional materials. First second harmonic generation and two-photon excitation microscopy is used to map the microscopic three-dimensional (3D) alignment, composition and distribution of the collagen and elastin fibers of periosteum, the soft tissue sheath bounding all nonarticular bone surfaces in our bodies. Then, using engineering rendering software to scale up this natural tissue fabric, as well as multidimensional weaving algorithms, macroscopic tissue prototypes are created using a computer-controlled jacquard loom. The capacity to prototype scaled up architectures of natural fabrics provides a new avenue to create advanced functional materials.

Natural materials such as animal and plant tissues exhibit remarkable stimuli responsive (smart) and adaptive properties that emerge macroscopically from the anisotropic multicellular assembly and directional secretion of nanoscopic extracellular matrix proteins: in essence, the cells themselves “spin and weave” the tissue *in situ*^1^. While top-down engineering approaches have elucidated multiscale structure-function relationships in a variety of tissue types, bottom-up approaches enable the engineering of so-called “emergent properties”^2^,^3^. Here, emergence describes properties or patterns that arise from the putting together of simpler elements which in themselves do not exhibit similar properties or patterns^2^.

One bottom-up approach to engineer this “emergence” is to emulate nature’s paradigm for synthesizing smart tissue fabrics, through technological platforms that enable the weaving of multiscale, anisotropic biomaterials by cells themselves^1^. While perfect control of such stem cell behavior presents currently insurmountable hurdles, some inroads have been made in guiding the assembly of higher order tissue architectures using bottom-up approaches^3^. A second bottom-up engineering approach to engineer emergence involves translation of nature’s weaving algorithms to weave scaled-up, multidimensional fabric architectures emulating natural fabric organization. Fundamental science and engineering approaches, including parametric scaling and similitude analysis, enable study of mechanics in systems of such small or large scale where direct experimental measures would be impossible^4^,^5^,^6^,^7^,^8^,^9^,^10^,^11^,^12^, *e.g.* the elastin and collagen weave of fibers making up the periosteum. Scaling and similitude approaches are commonly used in aerodynamics to understand behavior of large objects such as airplanes and autos on scaled down models in wind tunnels, where experimental conditions can be tightly controlled and experimental measures are feasible. For example, scaled-up, rapid prototyped models of complex cellular networks recently have been implemented to measure tissue permeability^12^. Here we present a novel approach to weave scaled-up tissue architectures modeled on one of nature’s own fabrics, the periosteum.

The periosteum is a *circa* 500 μm soft tissue sleeve composite, comprising an anisotropic elastin and collagen weave, which envelops every nonarticular bone surface of our bodies. The periosteum serves as an interface between bone and muscles and thus as a gatekeeper for transfer of molecular and physical (stresses, strains) information between the two tissue compartments^13^,^14^. Like Velcro, collagen containing Sharpey’s fibers anchor periosteum to bone, serving as a link between the external musculature and the internal skeleton^1^,^15^. Due to the distribution and intrinsic strength of Sharpey’s fibers, periosteum remains attached to bone even after significant trauma^15^. In addition, periosteum exhibits multiscale composite structure and viscoelastic properties^16^. Remarkably this smart **soft** tissue increases the failure strength (load at failure) and stability (maintenance of structure, alignment) during fractures of **hard** bone^15^,^16^. Furthermore, periosteum exhibits direction dependent (muscle <=> bone) and flow rate dependent permeability as well as mechanical properties, making it more permeable and stronger under impact loads^1^,^2^,^15^,^16^,^17^.

Here we aim to understand the inherent functional capacity of the periosteum by investigating its architecture, *i.e.* multiscale 3D distribution of its collagen and elastin fibers, via second harmonic generation and two-photon excitation microscopy (Fig. 1). We then test the feasibility of rendering these natural tissue weaves using computer-aided design software (Fig. 2) and scaling up nature’s patterns to weave periosteum-inspired, multidimensional fabrics with a jacquard loom (Fig. 3).

## Second Harmonic Imaging Microscopy (SHIM) of collagen and Two Photon Excitation Microscopy (TPEM) of elastin

SHIM has emerged as a robust tool to capture high-resolution, high-content, 3D representations of fibrillar collagen in live and *ex vivo* tissue without the need for exogenous labeling^18^,^19^. In SHIM, a frequency doubling of the incident light occurs in molecular structures that are repetitive and non-centrosymmetric^18^,^19^,^20^,^21^,^22^,^23^. The resulting coherently forward propagated waveform is not accompanied by a loss of energy nor is subject to photobleaching, making it an ideal technique to examine and quantify endogenous collagen functional textiles. Simultaneously, two-photon excitation microscopy can be used to image autofluorescent elastin, through reflected fluorescence^24^,^25^.

**Figure 1:**
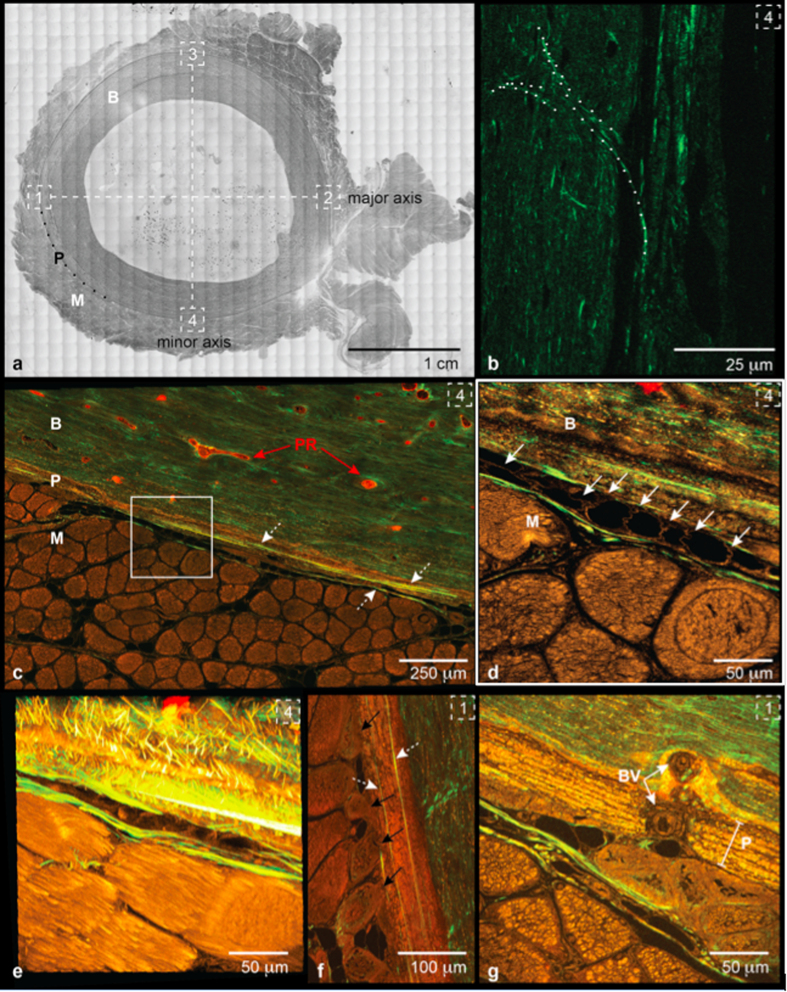
Periosteum as a woven interface between bone and muscle. (**a**) Transverse section of ovine femur diaphysis (B: bone, P: periosteum, M: muscle, dashed white squares indicate regions of interest, ROI, along the major (1, 2) and minor (3, 4) centroidal axes of bone, which are marked as dashed lines^28^,^29^). Periosteum and bone are ‘velcroed’ together by Sharpey’s fibers (**b**): outlined image of collagen arrangement making up two Sharpey’s fibers) that anchor the soft tissue sleeve to the hard surface of bone through a multitude of connections that distribute force without concentrating stresses. In turn, periosteum and muscle are physically connected by higher order architectures including springs (**c** - white square depicted in higher resolution in **d**, with springs highlighted by solid white arrows, *cf.* **Supplementary Animation 1**, Supplementary Figure 2, respectively, for additional dimension and higher resolution) and struts interwoven between the two (**f** & **Supplementary Animation 2**). (**c**) A 6 × 4 tiled image of the periosteum (P) bounded by bone (B) and skeletal muscle (M, muscle fascicles in cross section). Second harmonic signal from collagen fibers (dotted white arrows), shown in green; elastin signal shown in orange; Procion Red (PR) tracer delineating vascularization. ROI (white square) enlarged (**d**) to better visualize elastin fibers (indicated by arrows) and projected in 3D (**e**) from z-stack with 0.5 μm spacing in the z-direction. (**f**) Direct attachment of muscle, via struts, to the periosteum, and (**g**) blood vessel (BV) transecting the periosteum (P) (**Supplementary Animation 3**). (**b**–**g**) Numbered, dashed white boxes on upper right of each image correspond to ROIs along the major and minor axes depicted in (**a**).

Here, we first validate our combined SHIM-TPEM protocol on periosteum from sheep specimens prepared using standard protocols for undecalcified histology. Transverse sections were prepared from the ovine femoral diaphysis with intact surrounding musculature and vasculature stained *in vivo* using procion red *per* previous protocols^26^,^27^. Regions of interest (ROI) were imaged to highlight collagen and elastin fibers in adjacent musucloskeletal tissue compartments (bone, muscle, vasculature) and their respective microscopic structures along the major and minor axes of the diaphyseal shaft (Fig. 1a); these axes, calculated using an automated software, serve as objective indicators of tissue regions most and least able to resist bending forces in the axial plane^28^,^29^. For each ROI, a tiled image of the transverse (xy) plane, followed by a z-stack of one tile within the region, was captured to map in 3D space the composition and distribution of the collagen and elastin fibers as well as their higher order architectures.

The resulting merged tiled images demonstrate the high collagen content of bone (green) and high elastin content of muscle (brown) (Fig. 1). The thickness and composition of periosteum varies depending on proximity to nearby tissue compartments and structures such as muscle and vasculature. Where muscle or their fasciae adjoin to periosteum, the periosteum thickens, particularly in the posterior aspect. The periosteum and its neighboring muscle fasciae exhibit a varying composition and architecture of both collagen and elastin fibers. In some areas, fibers with a strong SHG signal, attributed to collagen, run along the bone’s circumference, though not spanning the entire ROI. These fibers represent components of periosteum’s fibrous layer, and their anisotropic arrangement likely underpins periosteum’s anisotropic mechanical reinforcement function and direction-dependent permeability^2^,^3^,^15^,^16^,^17^.

In other areas, collagen fibers insert at a slight angle directly into bone, indicative of Sharpey’s fiber structure and intrinsic function transducing force between muscle and bone (Fig. 1). The Sharpey’s collagen fiber bundles are thicker and emit a stronger signal than the collagen fibers within the periosteum sleeve composite. The thickness of the reflected fiber correlates to its contrast intensity, which varies as a function of the angle between the fiber and the laser beam^20^. Thus, for the first time to our knowledge, we were able to track in 3D space (in an approximately 40 μm thick z-stack) the angled collagen fibers making up single Sharpey’s fibers as they exit the periosteum and anchor into bone (Fig. 1b). Similarly, we also were able for the first time to visualize the interaction of the periosteum’s collagen fibers with neighboring muscle fascia and perimysium (Fig. 1c–g). Particularly in regions where muscle fascicles are in close association with the periosteum, collagen fibrils can be seen linking the periosteum to the perimysium, another indicator of direct force transfer (Fig. 1f). Though collagen is much less evident in the muscle *per se*, SHIM enables visualization of thin collagen fibers in the fasciae bordering muscle groups, perhaps providing mechanical support and stability during muscle distension. Based on these observations, we envision the muscle fascia as a continuous bounding layer of collagen fibers spanning superficial and deep muscle layers to the periosteum and finally, bone. In sum, the collagen fibers themselves exhibit different structures in different tissues, with loose and wavy collagen strands in muscle regions, to dense and straight bundles closer to the bone, and higher order architectures such as Sharpey’s fibers evident between tissue compartments.

Imaged in the transverse section of the long bone sample, the TPEM visualized elastin components of the periosteum resemble a dynamic layer enveloping bone. The elastin signal is also detected in muscle tissue structures, providing architectural information for fibers within muscle fascia, perimysium and blood vessels. With regards to muscle, distinct muscle groups can be seen connected via elastin coils resembling springs (Fig. 1d and **Supplementary Animation 1,2**) with other areas adhering directly to the periosteum (Fig. 1f and **Supplementary Animation 3**). In some ROIs multiple such coils formed a loosely woven webbed structure. These spatially varying features likely reflect the local mechanical environment of the tissue, *e.g.* elastin springs would maximize flexibility while providing elastic dampers during maximal muscle distension and spring back (Supplementary Fig. 1 and **Supplementary Animation 3**). Blood vessels, identified through the strong elastin signal of the vessel walls containing procion red filled channels, present abundantly in association with the periosteum (Fig. 1g and **Supplementary Animation 4**). Some blood vessels transect the periosteal layer to form the Volkmann canals, which insert into cortical bone and connect with the axially aligned Haversian channels. This multifunctional physiological tapestry comprises the fibrous weave of elastin and its higher order architecture into tissue fabric, bridging structures and vascular channels, highlighting the emergent structures which underlie the smart mechanical and permeability properties of the periosteum.

**Figure 2:**
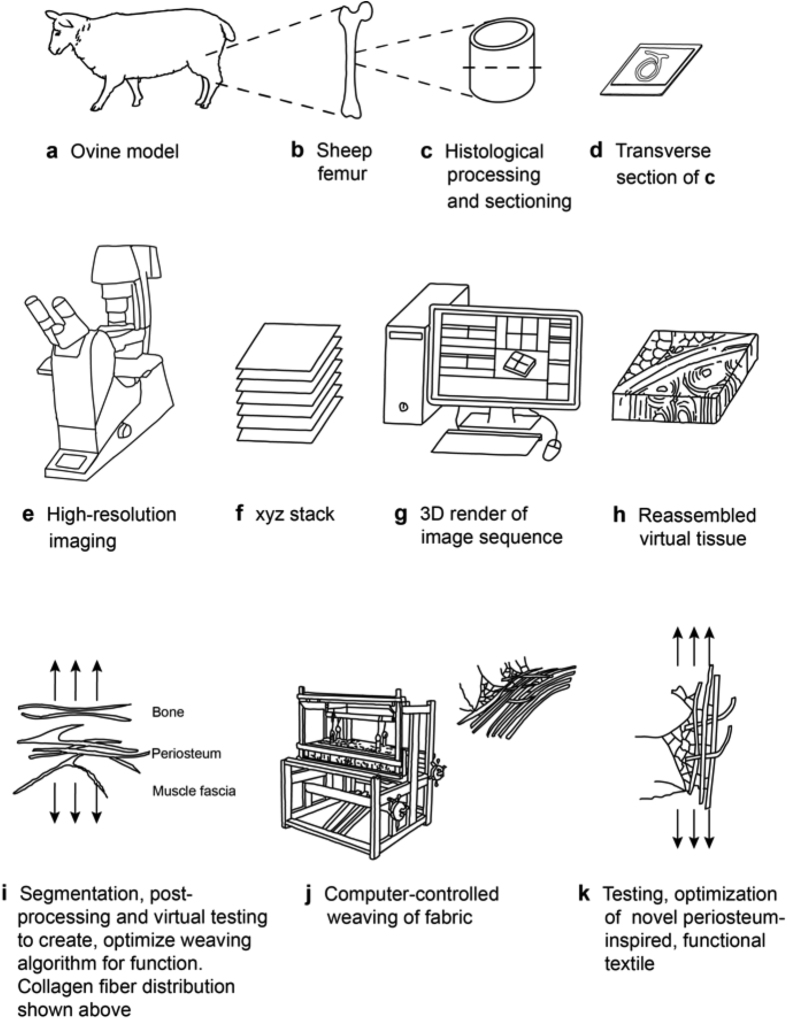
Schematic diagram illustrating the process for bottom-up weaving of multidimensional, natural tissue-inspired fabrics. (**a**–**d**) A transverse section of an ovine femur mid-diaphysis, with periosteum and surrounding muscle intact, stained with procion red solution. (**e,f**) Acquisition of high-resolution z-stacks using paired SHIM and TPEM imaging protocols to visualize structural proteins including collagen and elastin. (**g**–**i**) 3D rendering of z-stacks to convert image sequence into STL files for 3D computational modeling. (**j**) Conversion of virtual model into a physical model using multidimensional weaving technology; periosteum-inspired fabric shown above, right. (**k**) Optimization of novel functional textile via mechanical testing. J.L.N. created all schematic drawings depicted in this figure.

**Figure 3:**
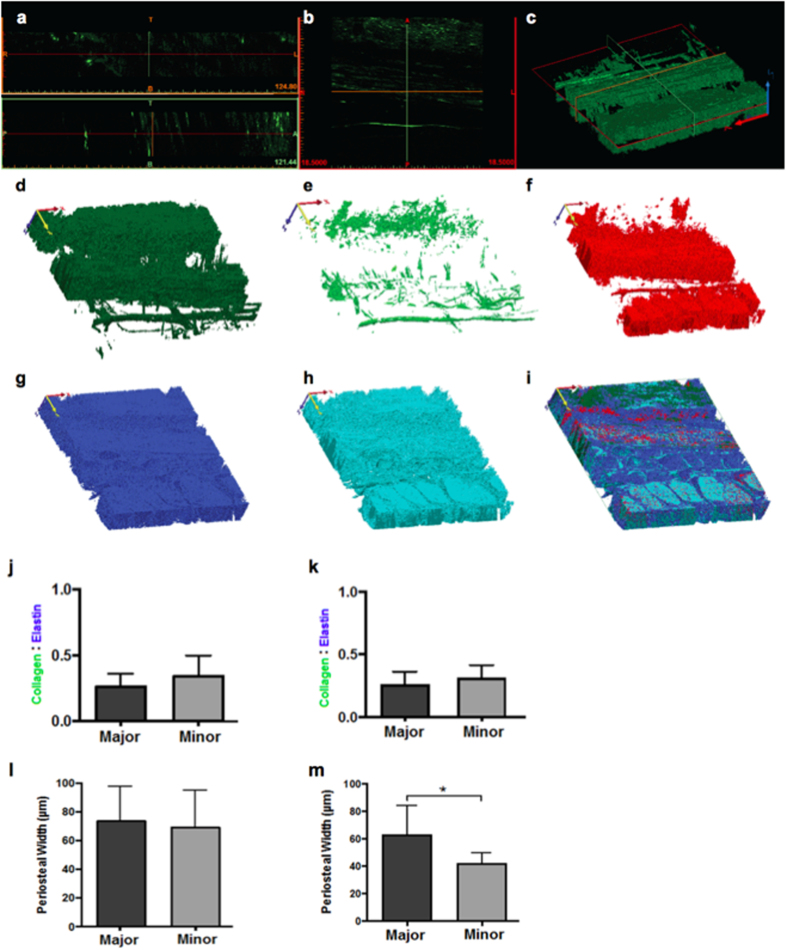
(**a**–**c**) 3D rendering of z-stacks (depth *circa* 40 μm) of SHG/collagen channel to STL files. 3D modeling of SHIM **(c)** together with TPEM and procion red channels comprising (**d**) weak collagen, (**e**) strong collagen, (**f**) vascularization, (**g**) weak elastin, (**h**) strong elastin signals, combined in (**i**), where *xyz* voxel size is 0.48 μm × 0.48 μm × 0.5 μm. **(j–m)** Ratio of collagen: elastin fibers across (**j**) different ovine specimens (n = 5) and (**k**) axially along same specimen (n = 3). Periosteal width across (**l**) different ovine specimens (n = 5) and (**m**) axially along the same specimen (n = 3).

Imaging the periosteum using SHIM and TPEM enables high resolution mapping of elastin and collagen fibers and their higher order architectures in context of surrounding tissue compartments. As a next step in our bottom-up approach we used the z-stacks from our novel microscopy protocol to create scaled up 3D models, which accurately represent the composition and spatial architecture of the image sequences and the tissue itself (Fig. 2). To achieve this, each channel in the z-stack was imported separately as an image sequence into Mimics^®^ for 3D rendering (Materialise, MIS v18.0 Beta) and then masked according to its signal intensity. The masks were converted to STL files and combined to create a composite 3D model comprising collagen, elastin and vascular components (Fig. 3). This model then served as a pattern template for a custom-configured jacquard-weaving algorithm (ArahWeave, arahne CAD/CAM for weaving) and for weaving of physical, scaled up prototypes (AVL Looms, Inc.).

**Figure 4:**
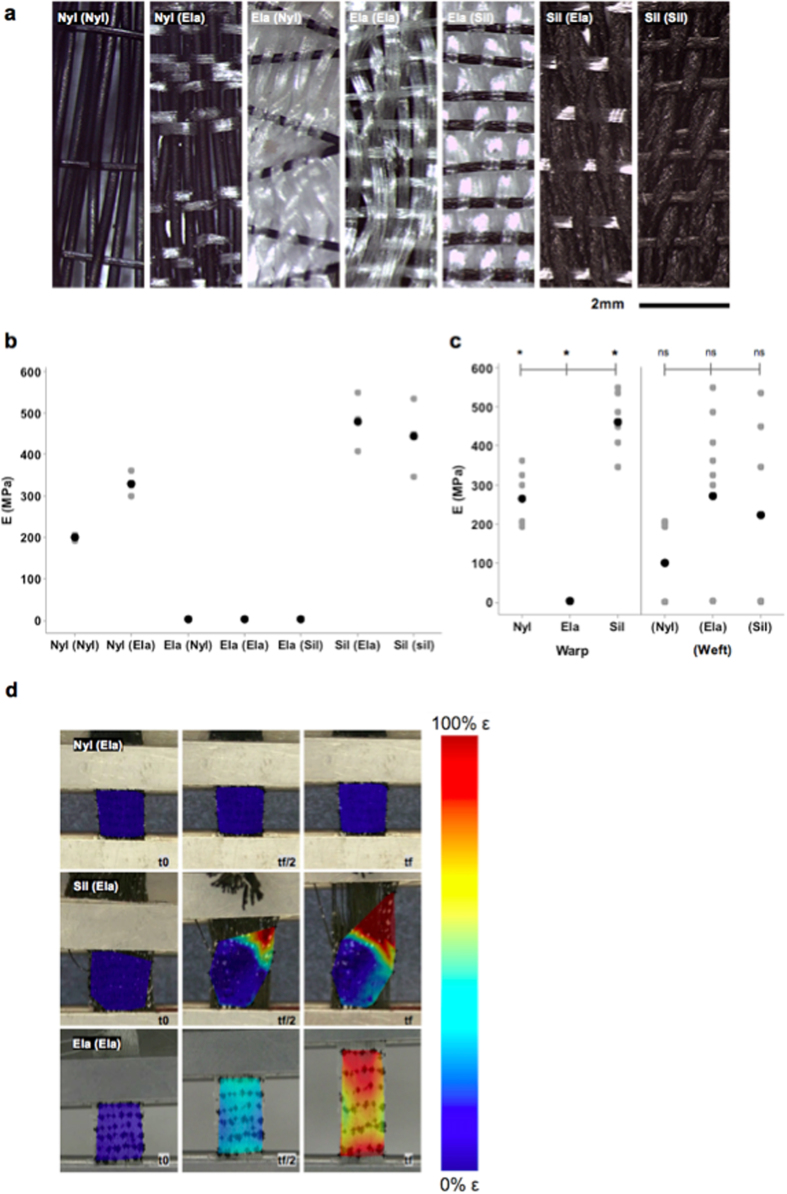
(**a**) Stereographs of woven swatches consisting of various warp and (weft) combinations (x12.5). **(b**,**c)** Individual value plot comparing elastic moduli of prototypes comprising different combinations, *i.e.* warp (weft), of nylon, elastane and silk (*p < 0.05). **(d)** Strain maps of prototypes at t_0_, t_f/2_, t_f_, where t = time, and f = final.

In a first test of the scaled-up tissue weaving concept^30^,^31^, a series of textile swatch prototypes were woven, using specific combinations of collagen (nylon monofilament and braided silk suture) and elastin (elastane yarn), in a twill pattern designed to idealize periosteum’s intrinsic weave (Fig. 4a). As shown by the mechanical testing of the resulting textile swatches, the swatches’ capacity to resist tension are orthogonally anisotropic and highly dependent on the warp yarn composition of the weave. The scaled up textile swatches exhibit emergent properties suggestive of periosteum’s natural collagen and elastin weave. For example, although nylon monofilament (Nyl) suture weaves exhibit a lower elastic modulus (264.5 ± 73.1 MPa) than braided silk suture (Sil) warp weaves (461.7 ± 77.5 MPa), the observed strain distributions in the materials indicate that nylon warp weaves are much more resistant to tension than equivalent silk weaves (Fig. 4b–d). In contrast to both nylon and silk weaves, elastane (Ela) warp weaves are highly elastic, and exhibit little resistance to tension (2.106 ± 0.419 MPa), with a ubiquitously uniform strain profile (Fig. 4d). Interestingly, the weft yarns of the elastane warp weaves show the capacity to modulate strain distribution during axial loading, perhaps due to their low elastic modulus. Taken together, these first tests on a series of textile swatch prototypes demonstrate the feasibility of using SHIM in combination with TPEM to develop and prototype structurally relevant, scaled-up woven prototypes mimicking periosteum’s sophisticated, complex and composite tissue fabric of as well as its smart stress-strain properties. Such scaled up, mechanically functional textiles lend themselves for use in the safety and transport sector. Ongoing studies are implementing these approaches at the microscale using engineered collagen and elastin and other biological structural proteins, for rapid implementation in the medical sector^30^,^31^.

**Figure 5:**
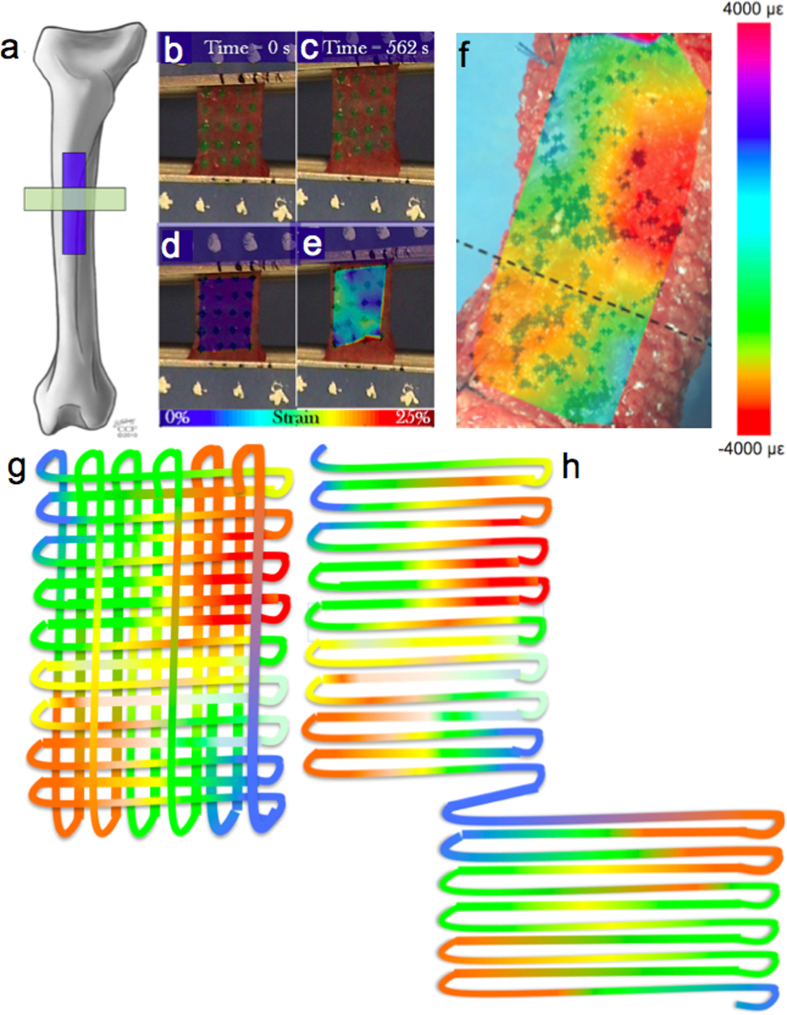
Recursive weaving and spinning concept. **(a**–**e)** Example of a tensile testing of anisotropic sheep femur periosteum samples, analogous to testing of swatches in Fig. 4. (**b,c**) DIC imaging of displacements under load at time zero and 562 seconds. (**d**,**e**) Strain mapping overlay on (**b**,**c**). Used with permission after^16^. (**f**) Testing of the highly complex, multidimensional fabric of periosteum *in situ* and *ex vivo*, under stance shift load. Heterogenous strain map at one point in time is depicted in color using digital image correlation and high resolution imaging (using high definition television lens). The dashed line in this view is orthogonal to the middiaphyseal imaging carried out using second harmonic imaging of collagen and elastin *per* Fig. 1(a), on the anterior aspect (corresponds to side of bone with ROI indicated by dashed square 3. Used with permission after^37^. **(g)** Schematic representation of fabric complexity showing weave of spatially and temporally varying threads, where the color scale depicts either local strains analogous to the endogenous tissue or, from an inverse perspective, local fiber stiffness. **(h)** Schematic depiction of recursive step where program algorithm is used to spin anisotropic, viscoelastic threads and to weave fabric. Adapted with permission^30^,^31^.

The acquisition of high-resolution, architecturally accurate 3D models enables rapid prototyping using computer-controlled weaving and/or other rapid prototyping modalities, allowing for automated and spatially consistent fabrication at high throughput. Other contemporary rapid prototyping techniques have been applied to manufacture tissue engineering scaffolds including electrospinning of nanoscaffolds^32^,^33^, 3D organ printing^34^ and integrative weaving of porous cartilage scaffolds^35^. Developments in electrospun nanofiber scaffolds enable the creation and manipulation of scaffolds at the cellular length scale, although this manufacturing process is not yet amenable to customization of the architecture, geometry and mechanical attributes needed to mimic the composite and sophisticated material properties of the periosteum or other similarly complex tissues. 3D printing offers distinct advantages with regard to flexibility in customizing geometries but is not yet effective for prototyping pieces with seamless mechanical gradients or parts that can withstand dynamic tension and bending. To date, integrative weaving has not yet captured the detailed fiber arrangement of biological tissues. Hence, to our knowledge, this study is the first of its kind, where natural woven architectures are mapped and replicated in scaled-up models to develop novel advanced materials and functional textiles. The only other potential way to replicate this process recursively would be to ‘unravel’ an inverse representation of the tissue mechanics as a stiffness map (Fig. 5), providing a pattern for the anisotropic weave, as shown above. Both approaches have been reduced to practice and the intellectual property has been protected (patent pending)^30^,^31^. Tests are underway to optimize the degree to which emergent properties are compromised by using the latter technique.

In summary, here we have demonstrated our novel protocol using SHIM in conjunction with TPEM to elucidate the organization and distribution of the collagen and elastin fibers of the periosteal sheath and to replicate nature’s smart properties through creation of advanced materials via scale up of tissue architectures and multifunctional weaving. Inspired by nature’s paradigms, this disruptive technology (defined as an “innovation that creates a new market and value network and eventually disrupts and dispaces an existing market and value network”^36^) has significant implications for the development of next-generation advanced materials and mechanically functional textiles, including biomedical materials and even materials in transport and safety industries.

## Methods

### Sample preparation

Ovine studies were approved by the Institutional Animal Care and Use Committee (IACUC) of the Canton of Grisons, Switzerland. All methods were carried out in accordance with the relevant guidelines and regulation of this IACUC. To visualize and quantify bone perfusion, 0.8% procion red solution was intravenously injected (Imperial Chemical, London) at a dosage 0.01 l/kg body mass 5 minutes prior to euthanasia^26^,^27^. Immediately after euthanasia, the femur, periosteum and surrounding muscle layer were resected and prepared for fixed, undecalcified histology using standardized protocols^27^. The resulting polymethylmethacrylate embedded tissue blocks were sectioned transversely, every 500 μm, using a diamond wire saw (Wells Model 4240). After polishing to *circa* 100 μm (Buehler Automet 2000 Polisher), sections were mounted on glass slides with glass coverslips (Eukitt). To relate tissue fabric organization to prevalent mechanical loading histories, the major and minor centroidal axes of the bone cross-sectional area^28^,^29^ were calculated using a macro in Image J (NIH Image J 2 v1.49). Major and minor centroidal axes (CA) represent cross-sectional geometric properties, *i.e.* the bending axes about which long bones are most (major CA) and least (minor CA) likely to resist a given bending load^28^. Prior studies showed that these reference points can be calculated in an automated way, reducing the possibility for bias while allowing for direct relation of outcome measures to loading patterns in age and treatment matched cohorts. The major and minor CAs were marked on the outer edges of the mounted bone specimens and served as reference points for high-resolution microscopy (Figs 1 and 3a–d).

### Imaging protocol

The specimens were imaged using a Leica SP5 II inverted microscope equipped with a Spectra Physics MaiTai HP DeepSea titanium sapphire multiphoton laser tuned to 830 nm (~100 fs pulse), an xyz high precision multipoint positioning stage and a 63 × 1.3NA glycerol objective. The forward propagated second harmonic collagen signal was collected in the transmitted Non-Descanned-Detector using a 390–440 nm bandpass filter. The two-photon excitation of elastin was excited at 830 nm and collected in the photo-multiplier tube (PMT) using a 435–495 nm emission filter. This filter was used to segment away autofluorescence that was observed in the green channel that did not completely correspond to elastin architecture. For the procion red signal, a 561 nm excitation was collected in the PMT using a 580–650 nm emission. A tiled scan was collected at the four quadrants correlating to the major and minor centroidal axes in the previously mentioned 3 channels, plus brightfield. Each 246 μm × 246 μm tile was imaged at 12-bit with a scan speed of 100 Hz and a resolution of 2.081 pixels per micron. Within the tiled area, a high-resolution xyz stack, with a step size of 0.5 μm and a voxel size of 0.48 μm × 0.48 μm × 0.50 μm, was acquired to capture the distribution of collagen, elastin and vasculature of the specimens in three dimensional space (Figs 1 and 3e,f).

### Post-processing and modeling

Both tiled images and xyz stacks were imported into Image J2 (NIH Image J2 v1.49), merged into a tiled overlapping image and converted into a.tif file. The image sequences from the xyz stacks were imported via separate channels into Mimics^®^ (Materialise MIS Research18.0 Beta). The thresholds of the masks were determined by the intensity of the signal. Two masks were used to distinguish between strong and weak collagen and elastin signals. The lower threshold of the ‘strong mask’ was determined by the minimal intensity required to enable a mask with contiguous pixels that represent the features of the specimen. The lower threshold of the ‘weak mask’ was determined by minimizing noise in the specimen. For rapid prototyping, the masks were converted to.stl files and imported into a STL visualization software (Materialise 3-matic Research v10.0 Beta). Noise was removed from the models by filtering small shells up to 1 μm^3^ then re-converted into Binary STL files. The channels were then combined to create a composite model of the collagen, elastin and vascular architecture, which is completely scalable for subsequent rapid prototyping using novel multidimensional weaving algorithms^30^,^31^ (Figs 2 and 3g–i).

### Multidimensional weaving of scaled-up tissue architectures based on nature’s paradigms

A recursive approach is used to prototype fabrics based on natural tissue weaves and fibers. Yarn fiber composition and stiffness gradients are achieved through scale up of natural fiber gradients and spinning. Weaves and higher order architectures are achieved using customized computer-aided weaving algorithms (ArahWeave) on a jacquard loom (AVL Looms) configured for complete control of every yarn woven into the fabric as well as through postprocessing after fabric weaving, analogous to posttranslational modification of extracellular matrix proteins^32^. Nylon monofilament and braided silk suture (Teleflex Medical, Wayne, PA), and elastane yarn (Madeira, Freiburg, Germany) were used, respectively, to mimic collagen and elastin fibers (Fig. 3j,k).

### Mechanical Testing and Digital Image Correlation

Woven textile prototype swatches were tested mechanically in tension to physiological displacement (12 mm range) at a constant strain rate (0.1 mm/s) (Electroforce 3200, Bose Corporation, Eden Prairie, MN). To generate the strain map, markers were traced on a Canon Legria HF G25 camcorder for the duration of loading (Canon Inc., TYO, JP). Digital imaging correlation strain maps were computed using a customized cpcorr function in MatLab (Mathworks Inc., Natick, MA)^16^,^37^. To test significance of differences in elastic moduli between materials, one-way ANOVA statistical analysis was completed in Minitab 17 (Minitab Inc., State College, PA) (Fig. 4).

## Additional Information

**How to cite this article**: Ng, J. L. *et al*. Scale-up of nature’s tissue weaving algorithms to engineer advanced functional materials. *Sci. Rep.* **7**, 40396; doi: 10.1038/srep40396 (2017).

**Publisher's note:** Springer Nature remains neutral with regard to jurisdictional claims in published maps and institutional affiliations.

## Supplementary Material

Supplementary Information

Supplementary Animation 1

Supplementary Animation 2

Supplementary Animation 3

Supplementary Animation 4
