# Supplementary material for: Scale-up of nature’s tissue weaving algorithms to engineer advanced functional materials
Source: Sci Rep. 2017 Jan 11;7:40396. doi: 10.1038/srep40396 (PMC5225443; doi:10.1038/srep40396)
Supplement: Supplementary Information [file srep40396-s1.pdf]

Supplementary Information

Scale-up of nature's tissue weaving algorithms to engineer advanced functional materials

Joanna L. Ng, Lillian E. Knothe, Renee M. Whan, Ulf Knothe, Melissa L. Knothe Tate

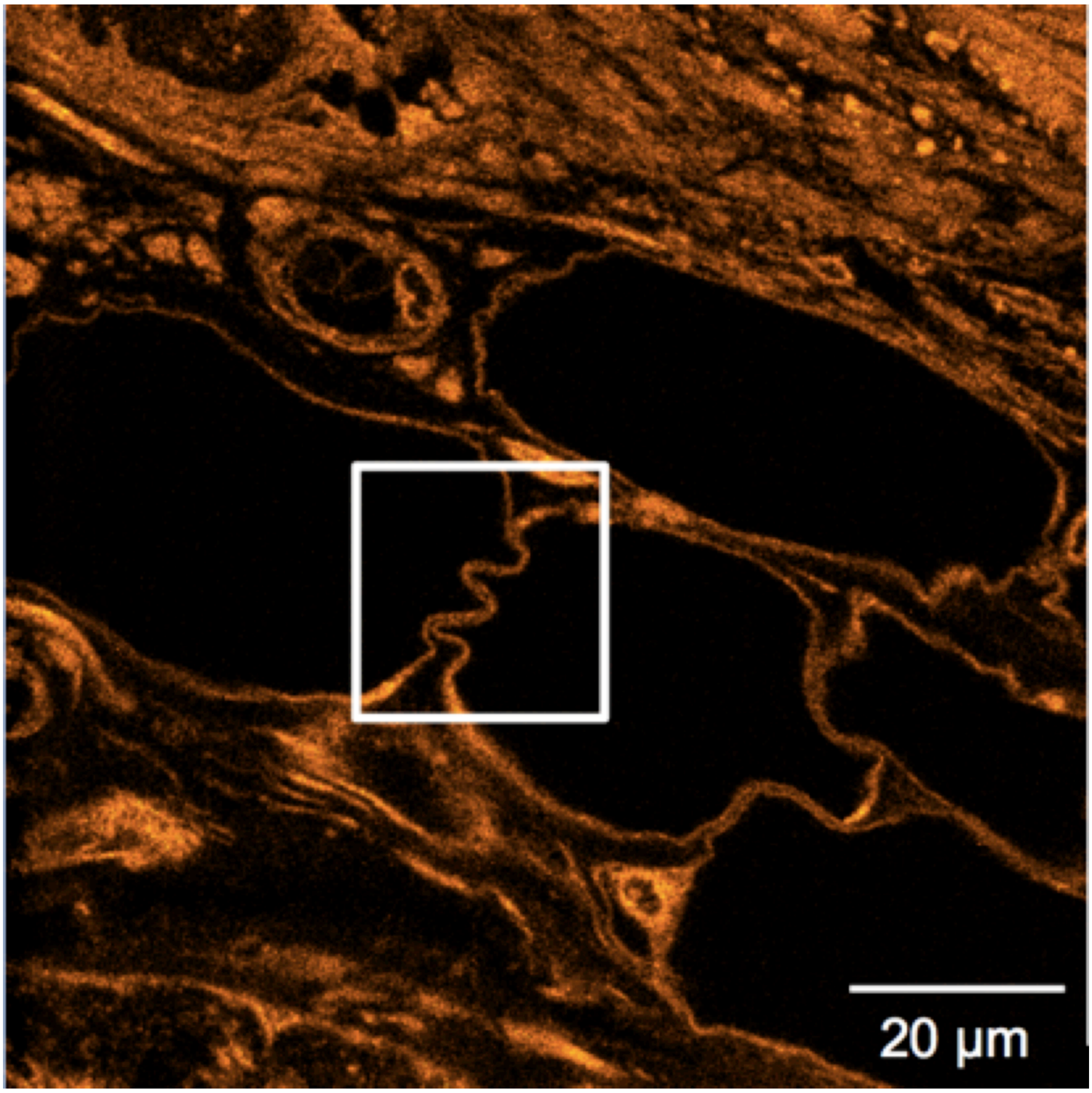

Supplementary Figure 1. Spring-like structures observed between periosteum and muscle (*cf.* **Supplementary Animation 1** for additional dimension).

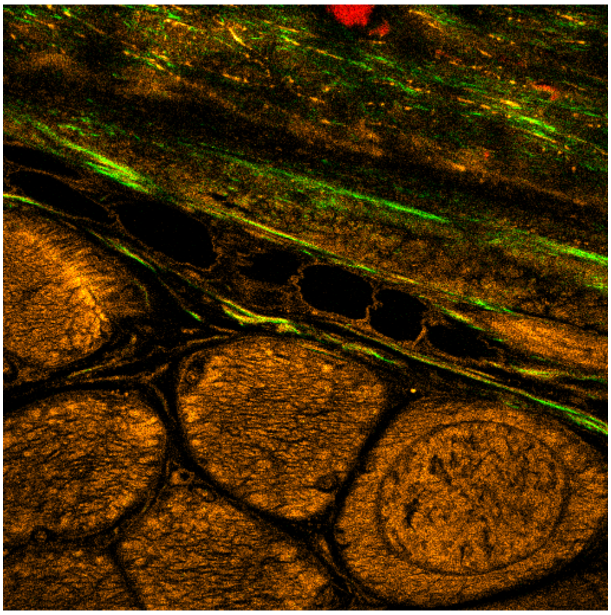

Supplementary Animation 1. Image of tiles (246 µm x 246 µm) showing SHG/collagen, elastin and procion red channels (quadrant 4).

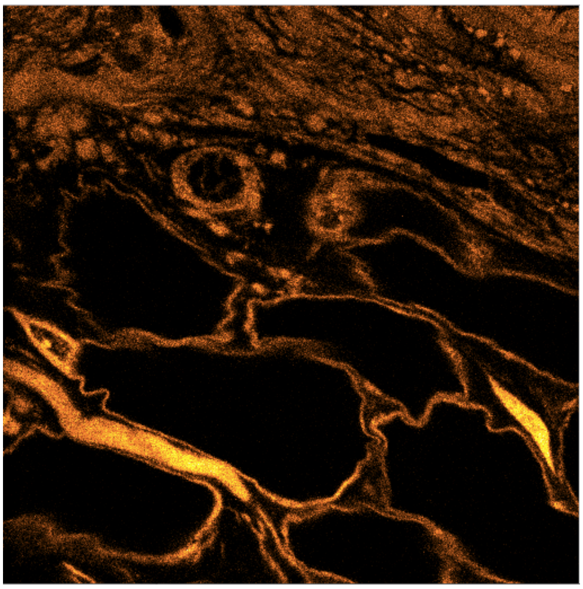

Supplementary Animation 2. Image of tiles (77 µm x 77 µm) depicting elastin in fascia between periosteum and muscle (quadrant 1).

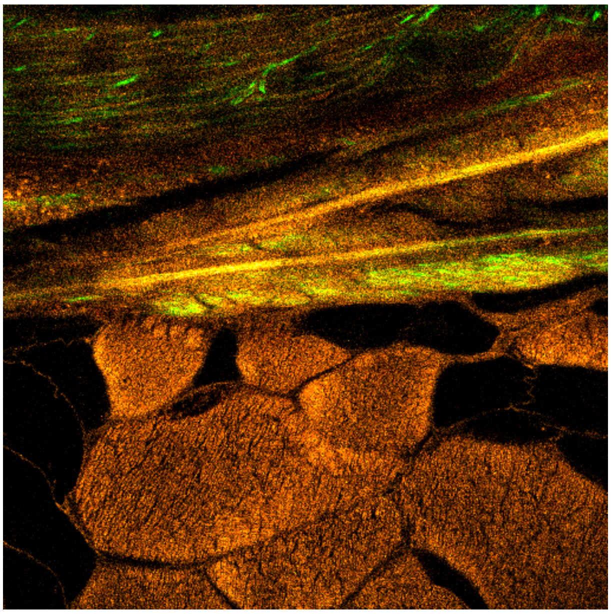

Supplementary Animation 3. Image of tiles (246 μm x 246 μm) showing SHG/collagen, elastin and procion red channels (quadrant 1). Here, muscle is directly attached to periosteum, hypothesized to be a means of force transfer across the two tissues.

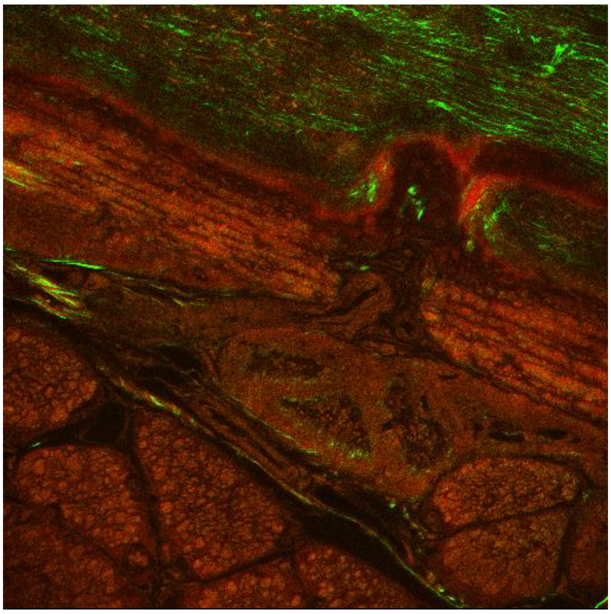

Supplementary Animation 4. Image of tiles (246 μm x 246 μm) showing SHG/collagen, elastin and procion red channels (quadrant 1).
