# Supplementary figures and images for: Scale-up of nature’s tissue weaving algorithms to engineer advanced functional materials
Source: Sci Rep. 2017 Jan 11;7:40396. doi: 10.1038/srep40396 (PMC5225443; doi:10.1038/srep40396)

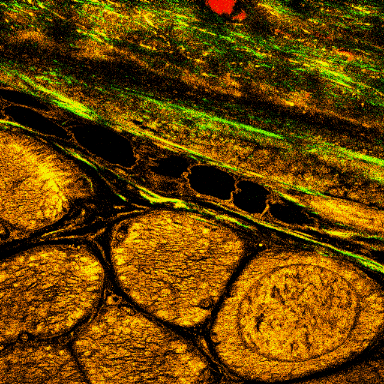

Supplement: Supplementary Animation 1 [file srep40396-s2.gif]

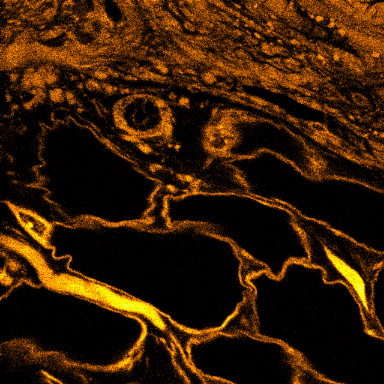

Supplement: Supplementary Animation 2 [file srep40396-s3.gif]

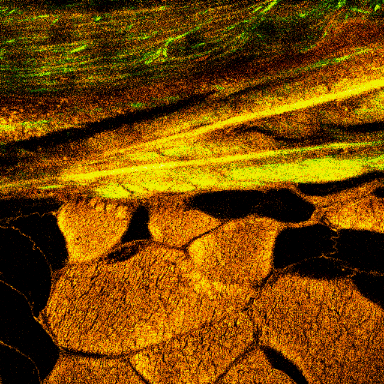

Supplement: Supplementary Animation 3 [file srep40396-s4.gif]

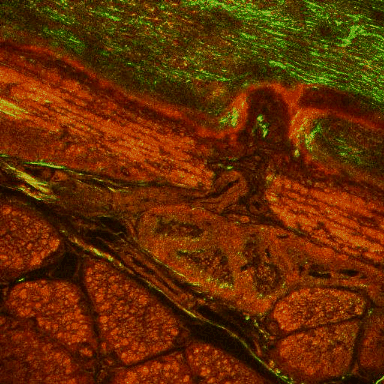

Supplement: Supplementary Animation 4 [file srep40396-s5.gif]
